# On the stability of queues with the dropping function

**DOI:** 10.1371/journal.pone.0259186

**Published:** 2021-11-03

**Authors:** Andrzej Chydzinski

**Affiliations:** Department of Computer Networks and Systems, Silesian University of Technology, Gliwice, Poland; LUMSA: Libera Universita Maria Santissima Assunta, ITALY

## Abstract

In this paper, the stability of the queueing system with the dropping function is studied. In such system, every incoming job may be dropped randomly, with the probability being a function of the queue length. The main objective of the work is to find an easy to use condition, sufficient for the instability of the system, under assumption of Poisson arrivals and general service time distribution. Such condition is found and proven using a boundary for the dropping function and analysis of the embedded Markov chain. Applicability of the proven condition is demonstrated on several examples of dropping functions. Additionally, its correctness is confirmed using a discrete-event simulator.

## Introduction

We study the classic queueing model with a single server, which additionally exploits a function $d:N_{0}\to \left[0,1\right]$, assigning the probability of dropping an arriving job to the length of the queue upon the new arrival. Function *d* is called the dropping function.

The well-known area of applications of queueing models with the dropping function is management of packet buffers in TCP/IP networks. Several types of mathematical functions have been analyzed as candidates for the dropping function. The list begins with the simple linear function [1], goes through the doubly linear [2], quadratic [3], cubic [4], the exponential one, [5], and a composition of linear and cubic functions [6]. Recently, a product of a linear function with its logarithm has been considered, [7].

The reason for applying the dropping function in TCP/IP networks is that classic finite-buffer queues cause many negative phenomenons, if used at routers output interfaces. These phenomenons include excessively long queues and delays (bufferbloat), unfair bandwidth sharing among the flows or even a full lock-out of some flows, flow rates synchronization, and other (see [8–11]).

As a remedy, preventive random dropping of arriving packets is recommended by the networking community, including the Internet Engineering Task Force, [8]. Depending on the method, the dropping probability can be calculated using more or less advanced algorithms, based on a few current or past system characteristics, [12–20]. An important class of such algorithms uses the aforementioned dropping function, i.e. assigning the dropping probability to the queue size, as in the mentioned works [1–6]. Queue management with the dropping function is easy to implement in networking devices and works sufficiently well, much better than the classic drop-tail queueing.

Queueing models with the dropping function can be used not only in networking. In everyday life, a decision of leaving the system before joining the queue may be induced by customers or other autonomous entities. For instance, some drivers may leave the highway when informed about the jam ahead of them, depending on the length of the jam. Perhaps, the longer the jam, the higher the probability of leaving the highway. In other systems, it may be the operator of the queue who decides about rejection of customers. In such systems, the dropping function is arguably the easiest mean of control of the performance of the queue. By shaping the dropping function it is possible, for instance, to obtain an arbitrarily small value of the average queue size, [21]. This may come at the cost of deterioration of some other characteristics, but can still be profitable, if these other characteristics are less important in the particular application.

From the mathematical perspective, it makes no sense to restrict the model to one type of the dropping function only. Therefore, we do not assume here any special type of the dropping function and the results are applicable to all of them, including those mentioned above.

Moreover, an infinite buffer is assumed herein. In this way, the considered model generalizes several classic single-server models, both with finite and infinite buffers. For instance, assuming *d*(*n*)≡0, we get the classic queueing model with unlimited buffer. If *d*(*M*) = 1 for some *M* and *d*(*n*) = 0 for *n* < *M*, then we obtain the classic model with a finite buffer of size *M*. Namely, in the classic queueing system with a buffer of size *M*, a job arriving when the buffer is full is dropped. This happens if and only if the queue size is equal to *M*. The job is accepted if and only if the queue size is less than *M*. On the other hand, in a queueing system with the dropping function *d*(*n*) such that *d*(*M*) = 1 and *d*(*n*) = 0 for *n* < *M*, an arriving job is also dropped if and only if the queue size is equal to *M*, and accepted if and only if the queue size is less than *M*. This follows directly from the definition of the system with the dropping function. Thus the systems of both types operate in exactly the same way.

When dealing with an infinite-buffer queue, the question of stability arises, where stability is understood as the existence of a proper limiting distribution of the queue length. Classic systems (no dropping function) are known to be stable if the system load, *ρ*, is less than 1, and unstable otherwise. A system with the dropping function is sometimes stable even if the load is greater than one, simply because an aggressive dropping function can decrease the load actually carried, in opposite to the load offered. For instance, consider a queueing system of M/D/1 type, with Poisson arrivals of rate λ = 2, constant service time equal to 1, and the dropping function $d\left(n\right)\equiv \frac{3}{4}$. We obviously have *ρ* = 2 in this system. Moreover, every Poisson arrival can be dropped with constant probability $\frac{3}{4}$. It is well known that such dropping scheme creates another Poisson process, so called thinned Poisson process, of rate $\lambda^{\prime }=\left(1-\frac{3}{4}\right)\lambda =\frac{1}{2}$. Therefore, the original system works exactly in the same way the classic M/D/1 system, with Poisson arrivals of rate λ′ and no dropping function. Moreover, the new system is stable, as $\rho^{\prime }=\lambda^{\prime }\cdot 1=\frac{1}{2}<1$, so the original system is also stable.

In more complicated parameterizations, however, stability depends in a complex way on the system load and the form of the dropping function, thus the usability of systems with the dropping function relies heavily on availability of tests for their instability. Therefore, the research question asked in this paper is whether there exists a simple, easy to check criterion for instability of a queue with the dropping function.

The main contribution of this paper is a condition sufficient for instability of a queueing system with the dropping function, found under assumptions of Poisson arrivals and the service time of the general type (condition (6)). To the best of the author’s knowledge, this condition is new and has not been published yet. It enables quick and easy testing of instability of most practically useful system parameterizations. In addition to the main contribution, several examples of unstable and stable systems are shown, as well as results of simulations, used to verify experimentally the stability of these examples.

Stability of systems with the dropping function has been so far investigated in [22–24]. In particular, the case of the simple, purely Markovian M/M/1 queue, has been dealt with in [22]. The obtained results include several criteria for the system stability and instability of this simple model. As for the non-Markovian queues, in [23] a condition sufficient for stability of the M/G/1 queue was shown, while in [24], the same condition for stability of the G/M/1 system was proven. Neither [23] nor [24] proposes conditions, which make the system unstable. Moreover, the proofs in [23, 24] rely on the renewal theory and exploit the existence of some well-defined renewal processes, which cannot be defined in unstable systems. Thus such approach cannot be used herein in proving instability. Instead, an analysis of the structure of the underlying Markov chain is performed here to show instability.

Finally, several papers have been published on queues with the dropping function and a buffer of finite size (see e.g. [21, 25–30]). In these papers, the analysis of stability is not carried out, as a finite buffer makes the system stable automatically. However, the infinite-buffer model is more general and applicable, as it incorporates the finite-buffer version, but not vice versa.

The remaining sections are structured in the following manner. Section 2 consists of a formal description of the model analyzed in the paper. In Section 3, the main theorem on instability is formulated and proven. Then, in Section 4, some general comments on the applicability of the proven theorem, together with the previously proven criterion of the system stability, are given. They are accompanied with sample parameterizations of stable and unstable systems. Moreover, a few hard cases are discussed, i.e. such that within the state of the art we cannot say, whether the system is stable, or not. In Section 5, experimental verification of the theoretical results, carried out using a discrete-event simulator, is presented. Finally, in Section 6 remarks concluding the article and some suggestions of future work are presented.

## 2 The model

We analyze herein the single-server queueing model, with an infinite buffer.

Namely, jobs arrive to the server (service station) according to the Poisson process of rate λ. If upon a job arrival there are no other jobs in the system, the arriving job is served immediately. If upon a job arrival there are some other jobs in the system, the arriving job joins the queue. The queue is formed and served in the arrival order, i.e. the job at the head of the queue is the first to be served, after the service of the currently served job is finished.

The service time distribution is not further specified, it can have any form. Its distribution function is denoted by *F*(*t*).

The infinite buffer assumption means that there is no limit on the queue size. The queue can get arbitrarily long during the operation of the system.

Additionally, the dropping function mechanism is implemented in the system. Namely, every incoming job can be rejected (or deleted) upon arrival with probability *d*(*n*), where *n* denotes the queue length upon this new arrival, including the service position, if occupied. A rejected job does not enter the queue. Instead, it leaves the system immediately without service and never returns. The function $d:N_{0}\to \left[0,1\right]$ is not specified, it can have an arbitrary form.

By *X*(*t*) we denote the queue length at *t*, and it includes the service position. We employ the convention that process *X*(*t*) is left-continuous, namely *X*(*t*) = *X*(*t*−).

The load of the queue is traditionally defined as:

$$\begin{array}{c} \rho =\bar{F}\lambda , \end{array}$$ (1)

where

$$\begin{array}{c} \bar{F}=\int_{0}^{\infty }tdF\left(t\right), \end{array}$$ (2)

is the average service time.

Note that the model considered herein is the same as the one studied in [23]. It is also similar to models studied in [21, 22, 24–30]. Namely in [22, 24], the service time distribution is exponential. Herein, the service time has a general, arbitrary distribution. In [21, 25–30] on the other hand, the model has a finite buffer. Herein, an infinite buffer is considered. As argued in the previous section, a model with an infinite buffer is more general.

## 3 Instability of the queue

A single-server queue is said to be stable if and only if for every *n* = 0, 1, …, there exists a limit:

$$\begin{array}{c} \underset{t\to \infty }{\text{lim}}P\left(X\left(t\right)=n\right)=p_{n}, \end{array}$$ (3)

and it holds:

$$\begin{array}{c} \sum_{n=0}^{\infty }p_{n}=1, \end{array}$$ (4)

where P denotes probability. If either (3) does not exist for some *n*, or (4) does not converge to 1, the queue is said to be unstable.

**Theorem 1**. *If*

$$\begin{array}{c} d(n)<1,\;\;\;\;n\geq 0, \end{array}$$ (5)

and

$$\begin{array}{c} b=\underset{n\to \infty }{\text{lim inf}}\left[1-d\left(n\right)\right]\rho >1, \end{array}$$ (6)

*then the system with the dropping function d*(*n*) *is unstable*.

The general scheme of the formal proof, given below, is the following. Firstly, using assumption (6), a new dropping function, d*(*n*), is found. It has a very simple form and is more aggressive than the original *d*(*n*), in the sense that d*(*n*) > *d*(*n*) for every *n* greater than some *K*. This function is, however, carefully chosen and not too aggressive, so that it has a special property: if we replace the original *d*(*n*) in the system by d*(*n*), then the embedded Markov chain of the queueing system is transient. This fact can be shown using the simple, constant form of d*(*n*). Now, from the fact that the embedded Markov chain for the system with d*(*n*) is transient, and the fact that d*(*n*)>*d*(*n*), *n* > *K*, it follows that the embedded Markov chain for the system with *d*(*n*) must be transient as well. If the embedded chain for the original system is transient, then for every *n* the limiting probability of the queue size is zero, and the sum in (4) is zero as well, so the system is unstable.

P r o o f of Theorem 1. Without loss of generality it can be assumed that *t* = 0 is a departure epoch.

Let *X*_0_ = 0 and *X*_*n*_, *n* = 1, 2, … denote the queue length left behind by the *n*-th job leaving the system. The memoryless property of the interarrival time distribution yields that *X*_*n*_ constitute a Markov chain. It is easy to see that transition probabilities of this chain have the form:

$$\begin{array}{c} p_{ij}=P\left(X_{n+1}=j|X_{n}=i\right)=\{\begin{array}{ccc} \int_{0}^{\infty }Q_{1,j}\left(u\right)dF\left(u\right), & \text{if} & i=0,j\geq 0,\\ \int_{0}^{\infty }Q_{i,j-i+1}\left(u\right)dF\left(u\right), & \text{if} & i\geq 1,j\geq i-1,\\ 0, & & \text{otherwise}, \end{array} \end{array}$$ (7)

where *Q*_*n*,*k*_(*u*) is the probability that in an interval of length *u*, *k* jobs are allowed to the system, if initially the queue size was *n* and there were no departures in the considered interval.

The function *Q*_*n*,*k*_(*u*) can be perceived as the counting function of the arrival process thinned by the dropping function. It has been proven in [27] that:

$$\begin{array}{c} q_{n,k}\left(s\right)=\int_{0}^{\infty }e^{-su}Q_{n,k}\left(u\right)du=\frac{\prod_{j=0}^{k-1}\left[\lambda -\lambda d\left(n+j\right)\right]}{\prod_{j=0}^{k}\left[\lambda -\lambda d\left(n+j\right)+s\right]}. \end{array}$$ (8)

From (6), it follows that there exists such a natural number *K* that it holds:

$$\begin{array}{c} 1<\frac{1}{2}\left(b+1\right)<\left[1-d\left(n\right)\right]\rho ,\;\;\;\;n>K. \end{array}$$ (9)

We will show that the state *K* + 1 of chain *X*_*n*_ is transient, i.e. starting from this state, with a non-zero probability the chain will never get back to it.

To accomplish this, let us consider a new M/G/1 queueing system, with the dropping function d* defined as:

$$\begin{array}{c} d^{*}\left(n\right)\equiv 1-w, \end{array}$$ (10)

where

$$\begin{array}{c} w=\frac{b+1}{2\rho }. \end{array}$$ (11)

Obviously, (5), (9) and (10) yield:

$$\begin{array}{c} d\left(n\right)<d^{*}\left(n\right)<1,\;\;\;\;\;\;n>K. \end{array}$$ (12)

Let $X_{n}^{*}$ denote the queue length left behind by the *n*-th leaving job, in the new system. Obviously, $X_{n}^{*}$ is also a Markov chain. Moreover, it is known that a Poisson process thinned with a constant probability is another Poisson process. Thus $X_{n}^{*}$ is in fact a Markov chain associated with the classic M/G/1 queue (without the dropping function), with the arrival rate *wλ*.

Transition probabilities of chain $X_{n}^{*}$ have the form:

$$\begin{array}{c} p_{ij}^{*}=P\left(X_{n+1}^{*}=j\;|\;X_{n}^{*}=i\right)=\{\begin{array}{cc} \int_{0}^{\infty }\frac{e^{-\lambda }wt{\left(\lambda wt\right)}^{j}}{j!}dF\left(t\right), & \;\text{if}\;\;\;\;i=0,\;0\leq j,\\ \int_{0}^{\infty }\frac{e^{-\lambda }wt{\left(\lambda wt\right)}^{j-i+1}}{(j-i+1)!}dF\left(t\right), & \;\text{if}\;\;\;\;1\leq i\leq j+1,\\ 0, & \;\text{otherwise}, \end{array} \end{array}$$ (13)

while the load of the new queue is:

$$\begin{array}{c} \rho^{*}=w\lambda \bar{F}=w\rho =\frac{b+1}{2}>1. \end{array}$$ (14)

It is known that under condition ρ* > 1 chain $X_{n}^{*}$ is transient (see e.g. [31], page 237). As a consequence, with a positive probability q*, it will never get to any state *k* such that *k* ≤ *K*+1, starting from state *K*+1. Namely, we have:

$$\begin{array}{c} P\left(X_{1}^{*}>K+1,X_{2}^{*}>K+1,X_{3}^{*}>K+1,\ldots |X_{0}^{*}=K+1\right)=q^{*}>0. \end{array}$$ (15)

Therefore, with a probability *q* ≥ q* the chain *X*_*n*_ will also never get to any state *k* such that *k* ≤ *K* + 1, namely:

$$\begin{array}{c} P\left(X_{1}>K+1,X_{2}>K+1,\ldots |X_{0}=K+1\right)=q\geq q^{*}>0. \end{array}$$ (16)

The latter is a consequence of (12), i.e. the fact that for queues larger than *K*, the arrival process in the new system is thinned with a more aggressive dropping function, d*, compared with the original dropping function, *d*.

Therefore, we have proven that *K*+1 is a transient state of chain *X*_*n*_.

It is easy to see that under condition (5), chain *X*_*n*_ is irreducible, i.e. all its states communicate. Moreover, from (7) it follows that *p*_*jj*_ > 0 for every *j*, which means that the chain is aperiodic.

It is known that in an irreducible and aperiodic Markov chain all states are transient if just one of them is transient (see e.g. [31], p. 18). Thus every state *j* ≥ 0 is a transient state of chain *X*_*n*_ and we have:

$$\begin{array}{c} \underset{n\to \infty }{\text{lim}}P\left(X_{n}=j\right)=0,\;\;\;j\geq 0. \end{array}$$ (17)

As a consequence:

$$\begin{array}{c} p_{j}=\underset{t\to \infty }{\text{lim}}P\left(X\left(t\right)=j\right)=0,\;\;\;j\geq 0, \end{array}$$ (18)

which is in conflict with (4). This completes the proof of the theorem.

## 4 Discussion and examples

Firstly, we may notice that (5) is not a technical assumption simplifying the proof, but it is truly needed for the system instability. Indeed, if *d*(*M*) = 1 for some *M*, then the queueing system of interest is equivalent to a finite-buffer system with the buffer of capacity *M*, which is known to be stable, no matter with or without the dropping function.

Secondly, we did not assume in the proof of Theorem 1 that $\bar{F}$ is finite. Therefore, the theorem holds in the case $\bar{F}=\infty$ as well. Note that to meet the theorem assumptions in such case, it must hold *d*(*n*) < 1 for every *n* and *b* > 1, which then means that lim inf_*n*→∞_(1 − *d*(*n*)) > 0.

Now, in [23] it was shown that an M/G/1 queue with the dropping function *d*(*n*) is stable if

$$\begin{array}{c} \underset{n\to \infty }{\text{lim sup}}\left[1-d\left(n\right)\right]\rho <1. \end{array}$$ (19)

Therefore, in most cases the stability of an M/G/1 queue can be told by checking if one of criteria (6) or (19) is fulfilled. Namely, having a system with the dropping function, we can compute lim inf_*n*→∞_[1 − *d*(*n*)]*ρ* and lim sup_*n*→∞_[1 − *d*(*n*)]*ρ*. If the former is greater than 1, then the system is unstable. If the latter is smaller than 1, then the system is stable. (Note that both of them cannot hold simultaneously). Finally, if neither the former is greater than 1, nor the latter is smaller than 1, then the stability of the system is not settled, i.e. we cannot say, whether it is stable, or not.

For instance, assume any arbitrary service time distribution and *ρ* = 2. It is easy to check using criterion (6), that addition of one of the following dropping functions creates an unstable system:

$$\begin{array}{c} d_{1}\left(n\right)=\frac{1}{3}e^{-n}+\frac{1}{3}, \end{array}$$ (20)

$$\begin{array}{c} d_{2}\left(n\right)=\frac{1}{2\pi }\text{arctan}\left(\sqrt{n}\right), \end{array}$$ (21)

$$\begin{array}{c} d_{3}\left(n\right)=\frac{n-\text{log}(n+1)}{3n+1}, \end{array}$$ (22)

$$\begin{array}{c} d_{4}\left(n\right)=\frac{1}{{\left(n-3\right)}^{2}+2}, \end{array}$$ (23)

$$\begin{array}{c} d_{5}\left(n\right)=\frac{1}{5}{\text{sin}}^{2}\left(n\right), \end{array}$$ (24)

$$\begin{array}{c} d_{6}\left(n\right)=\frac{e^{n}\left|\text{cos}\left(n\right)\right|}{4e^{n}-n^{3}}. \end{array}$$ (25)

On the contrary, the following dropping functions make the system stable, based on criterion (19):

$$\begin{array}{c} d_{7}\left(n\right)=1-\frac{5}{n^{2}+5}, \end{array}$$ (26)

$$\begin{array}{c} d_{8}\left(n\right)=\frac{3n^{2}-\text{log}\left(n+1\right)}{4n^{2}+1}, \end{array}$$ (27)

$$\begin{array}{c} d_{9}\left(n\right)=\frac{1}{2\pi }\text{arctan}\left(n^{2}-10\right)+\frac{1}{2}, \end{array}$$ (28)

$$\begin{array}{c} d_{10}\left(n\right)=\frac{3-2e^{-{\left(n-6\right)}^{2}}}{4}, \end{array}$$ (29)

$$\begin{array}{c} d_{11}\left(n\right)=\frac{2n+\text{sin}(n)}{3n+1}, \end{array}$$ (30)

$$\begin{array}{c} d_{12}\left(n\right)=\{\begin{array}{cc} 0.9, & \text{if}\;\text{n}\;\text{is}\;\text{odd},\\ 0.6, & \text{if}\;\text{n}\;\text{is}\;\text{even}. \end{array} \end{array}$$ (31)

Note that *d*_1_ − *d*_3_ and *d*_7_ − *d*_9_ are monotonic for *n* ≥ 0, while *d*_4_−*d*_6_ and *d*_10_ − *d*_12_ are non-monotonic. Thus the monotonicity of the dropping function does not influence the stability of the system. It is just slightly easier to apply criteria (6) or (19) in the monotonic case, as in such case we have lim sup[1 − *d*(*n*)]*ρ* = lim inf[1 − *d*(*n*)]*ρ* = lim[1 − *d*(*n*)]*ρ*, so we can compute lim instead of lim sup and lim inf.

Now, when studying the stability of systems with the dropping function, the case with

$$\begin{array}{c} \underset{n\to \infty }{\text{lim}}\left[1-d\left(n\right)\right]\rho =1. \end{array}$$ (32)

is more difficult. In particular, in [22] it was shown that some systems fulfilling (32) are stable, while others are unstable. For instance, an M/M/1 system with *ρ* = 1 and the dropping function:

$$\begin{array}{c} d\left(n\right)=\frac{1}{\sqrt{n+2}} \end{array}$$ (33)

is stable, while with the dropping function:

$$\begin{array}{c} d\left(n\right)=\frac{1}{n+2} \end{array}$$ (34)

is unstable, while both meet (32). In other words, the limit equal to 1 in (32) does not provide sufficient information about the system stability and some additional criteria have to be used, which are so far unknown for the non-Markovian M/G/1 queue. They are known, however, for the simple M/M/1 queue (see [22], p. 4).

Finally, there are also systems, in which

$$\begin{array}{c} \underset{n\to \infty }{\text{lim sup}}\left[1-d\left(n\right)\right]\rho >1 \end{array}$$ (35)

and

$$\begin{array}{c} \underset{n\to \infty }{\text{lim inf}}\left[1-d\left(n\right)\right]\rho <1 \end{array}$$ (36)

hold. Consider, for example, a system with *ρ* = 2 and

$$\begin{array}{c} d\left(n\right)=\frac{\text{sin}(n)+1}{2}. \end{array}$$ (37)

The stability of such systems is also undecided within the state of the art.

## 5 Simulations

Validity of the presented theoretical results was also confirmed in simulations.

For this purpose, the Omnet++ simulation framework, [32], was exploited. Omnet++ is a modular framework, which consist of modules and messages exchanged between them. These messages can simulate occurrence of discrete events of any type. Therefore, Omnet++ can be used for simulating virtually all systems, which change their states in discrete moments in time. Typically, it is used for simulation of wired and wireless communication networks, queueing systems and networks, communication protocols, hardware architectures and the performance of complex software systems.

In our case, the simulation setup was rather simple. It consisted of three modules: the generator of packets, the server (service station) and the sink. In the generator, the simulated jobs were created. The time between the consecutive jobs was simulated to be exponentially distributed with parameter 1. This was accomplished by using the built-in *exponential(1)* random number generator.

The generated jobs were passed to the second module, the server. Upon entering, they were subject to filtering by the dropping function mechanism. Namely, depending on the current queue size *n*, the arriving job was allowed to the central module or deleted, with the probability 1 − *d*(*n*) and *d*(*n*), respectively. For storing the accepted jobs in the central module, a *cQueue* object was used, which is a useful queueing object in Omnet++, equipped with convenient methods for adding a job to the tail of the queue, removing a job from its head, reading the current queue size, etc. The jobs stored in the queue were served in the arrival order, i.e. a job from the head of the queue was the next to be served. The simulated service time was uniformly distributed in interval [0, 4], which was accomplished by using the built-in *uniform(0,4)* generator.

Finally, the served jobs were passed to the sink module, which simply deleted them one by one.

One simulation run lasted until some particular simulated time was reached, say *t* = 20. At this time, the current queue size was recorded in a text file and the simulation was terminated with *endSimulation()* function.

Such a simulation was then repeated 10000 times. Repeated execution is easy to carry out in Omnet++ using the *repeat* command in the configuration file.

In a result, a file containing 10000 queue sizes at time *t* = 20 was obtained. This file was then used to compute the average queue size and its standard deviation at time *t* = 20.

The whole process was then repeated using different values of *t*, in the range from 1 to 500, and different dropping functions, i.e. functions *d*_1_–*d*_12_ from Section 4.

At the beginning of each simulation run, the queue was empty, thus it was always *X*(0) = 0. The mentioned above distributions of the interarrvial and service time indicate that it was *ρ* = 2 in every case. According to the discussion presented in Section 4, under such assumptions the dropping functions *d*_1_-*d*_6_ should make the system unstable, while *d*_7_-*d*_12_ should make it stable.

**Fig 1:**
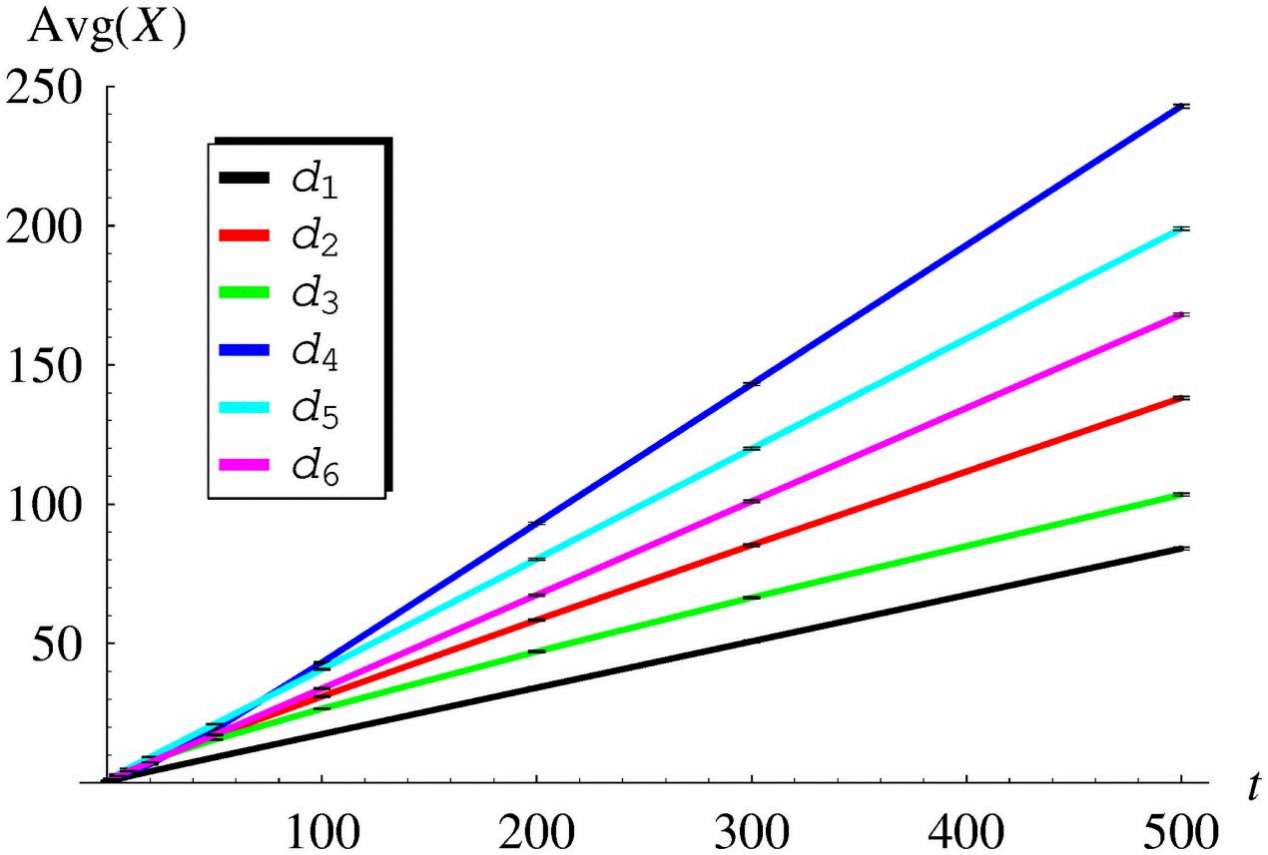
The average queue size versus time for dropping functions *d*_1_ − *d*_6_. The confidence intervals for the confidence level of 0.99 are shorter than the line width.

**Fig 2:**
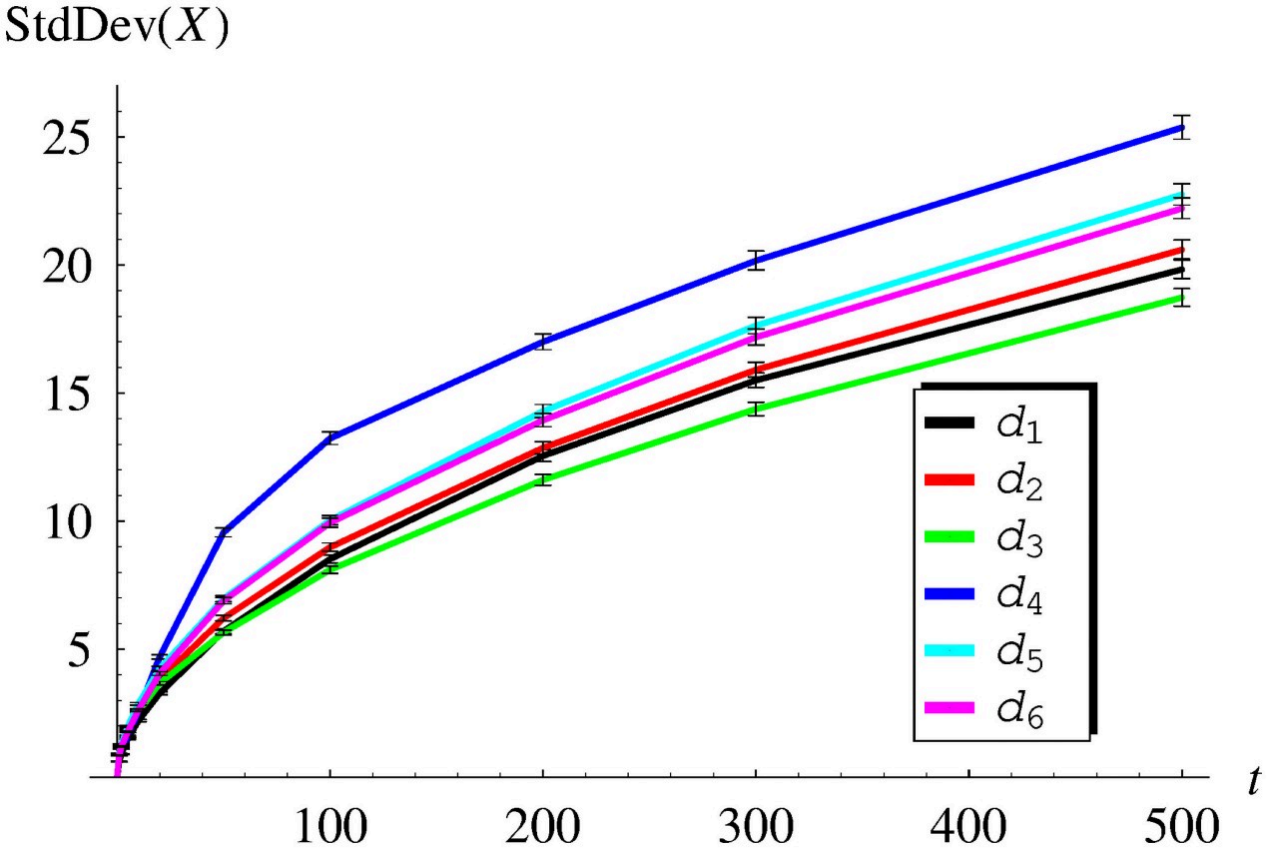
The standard deviation of the queue size versus time for dropping functions *d*_1_ − *d*_6_. The confidence intervals for the confidence level of 0.99 are added.

In Table 1 and Fig 1, the obtained average queue size versus time is shown for functions *d*_1_–*d*_6_. Similarly, in Table 2 and Fig 2, the standard deviation of the queue size is presented. As we can see, both the average value and the standard deviation grow with time.

**Table 1:** The average queue size at time *t* for dropping functions *d*_1_ − *d*_6_.

	*d*_1_	*d*_2_	*d*_3_	*d*_4_	*d*_5_	*d*_6_
*t* = 1	0.32	0.84	0.85	0.76	0.84	0.68
*t* = 2	0.62	1.52	1.56	1.34	1.53	1.28
*t* = 5	1.32	2.91	2.97	2.35	3.02	2.53
*t* = 10	2.29	4.75	4.79	3.64	5.15	4.17
*t* = 20	4.05	7.99	7.95	6.66	9.17	7.33
*t* = 50	9.13	16.93	15.49	19.07	21.03	17.18
*t* = 100	17.44	31.00	26.60	43.15	40.74	33.82
*t* = 200	34.14	58.42	47.09	93.15	80.31	67.42
*t* = 500	84.09	138.16	103.45	242.91	198.95	168.06

**Table 2:** The standard deviation of the queue size at time *t* for dropping functions *d*_1_ − *d*_6_.

	*d*_1_	*d*_2_	*d*_3_	*d*_4_	*d*_5_	*d*_6_
*t* = 1	0.61	0.91	0.92	0.85	0.91	0.86
*t* = 2	0.91	1.25	1.27	1.12	1.28	1.20
*t* = 5	1.50	1.86	1.84	1.60	1.98	1.78
*t* = 10	2.21	2.63	2.56	2.57	2.87	2.62
*t* = 20	3.28	3.82	3.67	4.70	4.24	4.05
*t* = 50	5.70	6.21	5.65	9.56	6.97	6.89
*t* = 100	8.52	8.98	8.10	13.23	10.03	9.94
*t* = 200	12.55	12.86	11.61	20.17	14.29	13.94
*t* = 500	19.83	20.60	18.73	25.37	22.75	22.21

However, note that in a stable system it must hold:

$$\begin{array}{c} \underset{t\to \infty }{\text{lim}}\text{Avg}(X(t))=\sum_{n=0}^{\infty }np_{n}=L=const, \end{array}$$ (38)

and

$$\begin{array}{c} \underset{t\to \infty }{\text{lim}}\text{StdDev}(X(t))=\sqrt{\sum_{n=0}^{\infty }{\left(n-L\right)}^{2}p_{n}}=D=const, \end{array}$$ (39)

which is a consequence of the fact, that in a stable system the limiting distribution {*p*_*n*_} is a proper distribution (see (3) and (4)).

Naturally, Avg(*X*(*t*)) and StdDev(*X*(*t*)) growing to infinity with time in Table 1, Fig 1 and Table 2, Fig 2, are in contrast with (38) and (39), respectively. Therefore, Tables 1 and 2 and Figs 1 and 2 present the behaviour of unstable systems. This confirms the conclusion obtained previously on the theoretical ground, that dropping functions *d*_1_-*d*_6_ make the system unstable.

**Fig 3:**
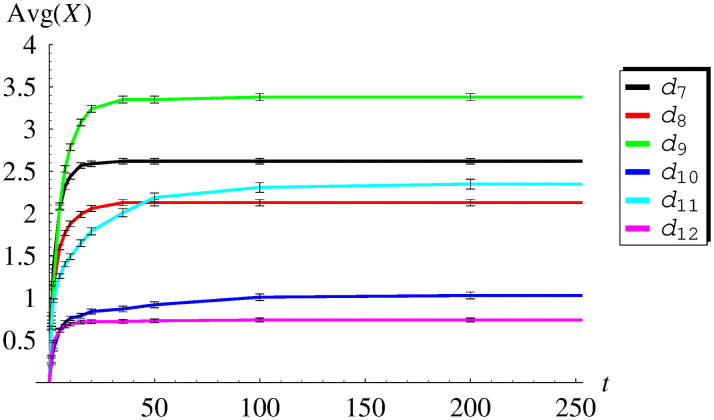
The average queue size versus time for dropping functions *d*_7_ − *d*_12_. The confidence intervals for the confidence level of 0.99 are added.

**Fig 4:**
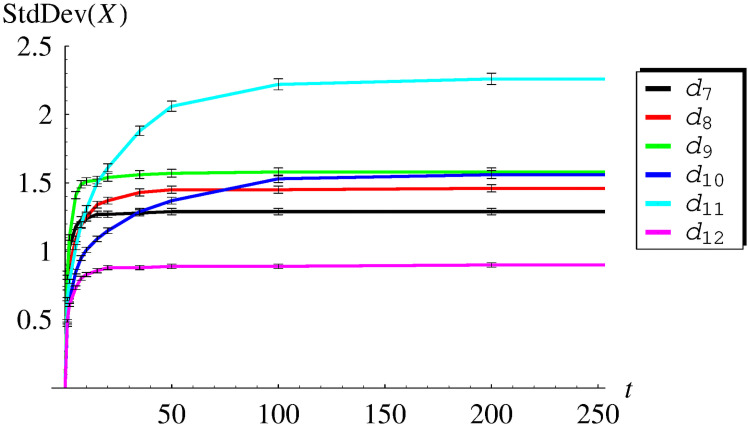
The standard deviation of the queue size versus time for dropping functions *d*_7_ − *d*_12_. The confidence intervals for the confidence level of 0.99 are added.

In Table 3 and Fig 3, the average queue size versus time is presented for functions *d*_7_–*d*_12_, while in Table 4 and Fig 4, the standard deviation is shown for the same set of dropping functions. As we can see, now Avg(*X*(*t*)) and StdDev(*X*(*t*)) converge in time to constant values, for every dropping function *d*_7_-*d*_12_. Such behaviour, consistent with (38) and (39), confirms the fact obtained previously on the theoretical ground, that all functions *d*_7_-*d*_12_ make the queueing system stable.

**Table 3:** The average queue size at time *t* for dropping functions *d*_7_ − *d*_12_.

	*d*_7_	*d*_8_	*d*_9_	*d*_10_	*d*_11_	*d*_12_
*t* = 1	0.81	0.72	0.64	0.22	0.65	0.30
*t* = 2	1.37	1.14	1.17	0.39	0.97	0.48
*t* = 5	2.07	1.60	2.09	0.62	1.26	0.61
*t* = 10	2.44	1.88	2.79	0.76	1.49	0.69
*t* = 20	2.59	2.06	3.24	0.84	1.79	0.72
*t* = 50	2.62	2.13	3.35	0.92	2.19	0.73
*t* = 100	2.62	2.13	3.38	1.01	2.31	0.74
*t* = 200	2.62	2.13	3.38	1.03	2.35	0.74
*t* = 500	2.62	2.13	3.38	1.03	2.35	0.74

**Table 4:** The standard deviation of the queue size at time *t* for dropping functions *d*_7_ − *d*_12_.

	*d*_7_	*d*_8_	*d*_9_	*d*_10_	*d*_11_	*d*_12_
*t* = 1	0.84	0.73	0.81	0.46	0.65	0.49
*t* = 2	1.03	0.89	1.10	0.64	0.78	0.60
*t* = 5	1.18	1.09	1.41	0.85	1.03	0.73
*t* = 10	1.24	1.25	1.49	1.01	1.31	0.83
*t* = 20	1.27	1.37	1.52	1.15	1.63	0.88
*t* = 50	1.29	1.45	1.57	1.37	2.08	0.89
*t* = 100	1.29	1.45	1.58	1.52	2.21	0.89
*t* = 200	1.29	1.46	1.58	1.56	2.26	0.90
*t* = 500	1.29	1.45	1.58	1.56	2.26	0.90

It is worth noticing, that in some cases the convergence to the steady state is much quicker than in others. For instance, in the case of *d*_7_, the system stabilizes at around *t* = 50, while in the case of *d*_11_, at around *t* = 200. This means that the form of the dropping function may have a deep impact on the convergence rate to the steady state.

Finally, there are confidence intervals marked in Figs 1–4. They were obtained for the high confidence level of 0.99, using the usual models of confidence intervals for the mean and standard deviation.

As we can see, these confidence intervals are practically invisible in Fig 1 (thinner than the line width) and barely visible in Figs 2–4 (slightly thicker than the line width). Therefore, the presented simulation results are very reliable from the statistical point of view. Such high statistical accuracy is a consequence of the fact, that each value of the average queue size and the standard deviation was obtained from a rather large number of simulations (10000).

## 6 Conclusions and future work

We have proven a condition sufficient for the instability of a queue with the dropping function, Poisson arrivals and general service time distribution, i.e. for the M/G/1 system in the Kendall’s notation, with the dropping function added.

Summarizing, we can now decide the stability of the queue either if

$$\underset{n\to \infty }{\text{lim inf}}\left[1-d\left(n\right)\right]\rho >1,$$

or

$$\underset{n\to \infty }{\text{lim sup}}\left[1-d\left(n\right)\right]\rho <1,$$

holds, no matter what is the form the dropping function, or what is the distribution of the service time. On the other hand, if

$$\underset{n\to \infty }{\text{lim inf}}\left[1-d\left(n\right)\right]\rho \leq 1,$$

and

$$\underset{n\to \infty }{\text{lim sup}}\left[1-d\left(n\right)\right]\rho \geq 1,$$

then the stability of the queue cannot be decided within the presented results. In particular, if

$$\underset{n\to \infty }{\text{lim}}\left[1-d\left(n\right)\right]\rho =1,$$

then the stability of the queue cannot be decided.

Twelve examples of stable and unstable systems were shown. Their stability or instability was first tested using the theoretical criteria given above, and then confirmed in simulations, performed using a discrete-event simulator Omnet++.

A few other important problems regarding stability of queues with the dropping function are still waiting to be solved.

First, a condition for instability of the G/M/1 system with the dropping function has to be proposed. It is likely, that (6) also holds for such systems, but a different, than presented here, proof is needed. This is due to the fact, that the embedded Markov chain has a different structure in the G/M/1 queue.

Second, the stability of the M/G/1 system with the dropping function may be further investigated. In particular, the hard problems with (32) or (35)–(32), could be studied. Perhaps one of the criteria of stability of the M/M/1 system with the dropping function can be expanded to incorporate the M/G/1 system as well. For instance, each of the following conditions:

$$\begin{array}{c} \underset{n\to \infty }{\text{lim}}n\frac{1-\rho [1-d(n)]}{\rho [1-d(n)]}>1, \end{array}$$ (40)

$$\begin{array}{c} \sum_{n=1}^{\infty }2^{n}\rho^{2^{n}}\prod_{k=1}^{2^{n}}\left[1-d\left(k-1\right)\right]<\infty , \end{array}$$ (41)

$$\begin{array}{c} \underset{n\to \infty }{\text{lim}}\rho \prod_{k=1}^{n}{\left[1-d(k-1)\right]}^{\frac{1}{n}}<1, \end{array}$$ (42)

is sufficient for the stability of the M/M/1 system, [22]. If extended to the M/G/1 case, they could be used to solve the hard problems with (32) or (35)–(36). Unfortunately, extending (40), (41) or (42) to the M/G/1 queue seems to be far from trivial.

Third, stability of systems with autocorrelated arrivals can be studied. Queueing systems with autocorrelated arrivals and the dropping function have been studied so far only in the case of the finite buffer and two selected arrival processes, namely the Markov-modulated Poisson process, [30], and the batch Markovian arrival process, [21]. In the case of the finite buffer, such systems are always stable. The infinite-buffer assumption changes this situation, and makes it necessary to use criteria for stability and instability, which are so far unknown for such arrival processes. Moreover, besides the aforementioned two processes, some other autocorrelated processes are used to model autocorrelation of arrivals in queueing systems, e.g. the fractional autoregressive integrated moving average (FARIMA) process. Stability conditions for queues with such arrival processes are also unknown.

Fourth, stability of queueing systems with the dropping function and continuous structure can be studied. For instance, in [29], a system with a buffer of continuous type is considered. In this case we say that the buffer has some volume. Moreover, each arriving job has a volume, i.e. a continuous size. Therefore, the domain of the dropping function is also continuous. The dropping function operates as follows: each arriving job is dropped with a probability depending on this job volume and the occupied volume of the buffer at the arrival time. In [29], the volume of the buffer is finite. Therefore, there is no need to deal with the stability of the system. In the case of the infinite buffer, the stability criteria should be found. They are so far unknown.

Finally, other special properties of the dropping function and their influence on the queueing system performance can be studied. For instance, in [33] it was shown using simulations, that a convex dropping function may be especially good for reduction of negative effects caused by packet losses. Perhaps this effect can be associated with special properties of convex functions (see e.g. [34]). Such effects can be further studied using formal analysis, instead of simulations.
